# Systematic extrahepatic Glissonean pedicle isolation for anatomical liver resection based on Laennec's capsule: proposal of a novel comprehensive surgical anatomy of the liver

**DOI:** 10.1002/jhbp.410

**Published:** 2017-02-03

**Authors:** Atsushi Sugioka, Yutaro Kato, Yoshinao Tanahashi

**Affiliations:** ^1^ Department of SurgeryFujita Health University1‐98 Dengakugakubo, KutsukakeToyoakeAichi470‐1192Japan

**Keywords:** Anatomy, Hepatectomy, Liver, Standardization

## Abstract

Anatomical liver resection with the Glissonean pedicle isolation is widely approved as an essential procedure for safety and curability. Especially, the extrahepatic Glissonean pedicle isolation without parenchymal destruction should be an ideal procedure. However, the surgical technique has not been standardized due to a lack of anatomical understanding. Herein, we proposed a novel comprehensive surgical anatomy of the liver based on Laennec's capsule that would give a theoretical background to the extrahepatic Glissonean pedicle isolation. Laennec's capsule is the proper membrane that covers not only the entire surface of the liver including the bare area but also the intrahepatic parenchyma surrounding the Glissonean pedicles. Consequently, there exists a gap between the Glissonean pedicle and Laennec's capsule that could be reached extrahepatically and allows us to isolate the extrahepatic Glissonean pedicle without parenchymal destruction systematically. For standardization, it is essential to approach the “six gates” indicated by the “four anatomical landmarks”: the Arantius plate, the umbilical plate, the cystic plate and the Glissonean pedicle of the caudate process (G1c). This novel anatomy would contribute to standardize the surgical techniques of the systematic extrahepatic Glissonean pedicle isolation for anatomical liver resection including laparoscopic or robotic liver resection and to bring innovative changes in hepatobiliary surgery for spreading safe and curable liver resection.

## Introduction

Anatomical liver resection with the Glissonean pedicle isolation is widely approved as an essential procedure for safety and curability 1, 2. Especially, the extrahepatic Glissonean pedicle isolation without parenchymal destruction should be an ideal procedure. Nevertheless, the surgical technique of the Glissonean pedicle isolation has not been standardized due to a lack of anatomical understanding. According to a rapid spread of liver resection including emerging minimally invasive liver resection such as laparoscopic and robotic surgery, it became increasingly imperative to standardize the surgical technique of the extrahepatic Glissonean pedicle isolation.

## Historical considerations about the Glissonean pedicle isolation

**Figure 1:**
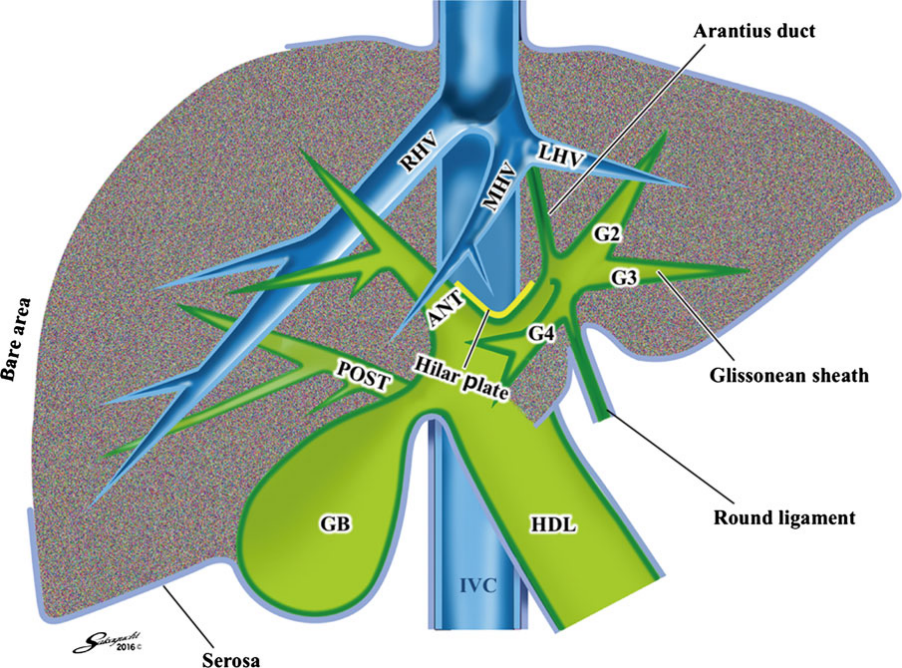
The schema of a conventional liver anatomy. According to a conventional liver anatomy, the surface of the liver is covered only by the serosa (*blue line*) and the bare area is covered by nothing. The cystic fossa is adjacent to the subserosal layer of the gallbladder. The Glissonean pedicle is also directly in contact with the intrahepatic parenchyma that means parenchymal destruction is inevitable during the Glissonean pedicle isolation. *ANT* right anterior Glissonean pedicle, *GB* gallbladder, *HDL* hepatoduodenal ligament, *IVC* inferior vena cava, *LHV* left hepatic vein, *MHV* middle hepatic vein, *POST* right posterior Glissonean pedicle, *RHV* right hepatic vein

Couinaud described the three different ways to approach the Glissonean pedicles 3: the intrafascial, the extrafascial, and the extrafascial and transfissural approach. The intrafascial approach means the individual vascular transection method, whereas the extrafascial approach means the Glissonean pedicle approach. The extrafascial approach is further divided into the intrahepatic and the extrahepatic approach according to the presence or absence of parenchymal destruction. The extrafascial and transfissural approach is the pedicle approach with major liver transection through the main portal or the umbilical fissure. Therefore, the extrafascial or the Glissonean pedicle approach could be subdivided into three categories as follows: the intrahepatic approach with minor liver transection, the intrahepatic with major liver transection and the extrahepatic approach without parenchymal destruction. Couinaud first proposed the intrahepatic Glissonean pedicle isolation with major liver transection in 1957 4. Afterwards, Lin et al. 5, Ton‐That‐Tung and Nguyen‐Duong‐Quang 6 and Okamoto 7 successfully performed the intrahepatic Glissonean pedicle isolation with major liver transection through the main portal fissure. Meanwhile, Galperin and Karagiulian described the intrahepatic approach with minor transection using digitoclasia in 1989 8 and Machado et al. reported the same type of approach using a clamp or a vascular stapler 9, 10. However, the intrahepatic isolation might be accompanied by a potential risk of unexpected injury to the portal pedicle or the hepatic vein. Therefore, the extrahepatic Glissonean pedicle isolation should be an ideal procedure. Couinaud first proposed the concept of the extrahepatic Glissonean pedicle approach for left hepatectomy in 1985 10, 11 and Takasaki et al. actually performed for the first time as the Glissonean pedicle transection method 12, 13. Although this is a well‐known method, a conventional liver anatomy shown in Figure 1 had been prevented standardization because parenchymal destruction was inevitable without a membrane on the opposite side of the Glissonean pedicles.

## Historical considerations about the surgical anatomy of the liver

**Figure 2:**
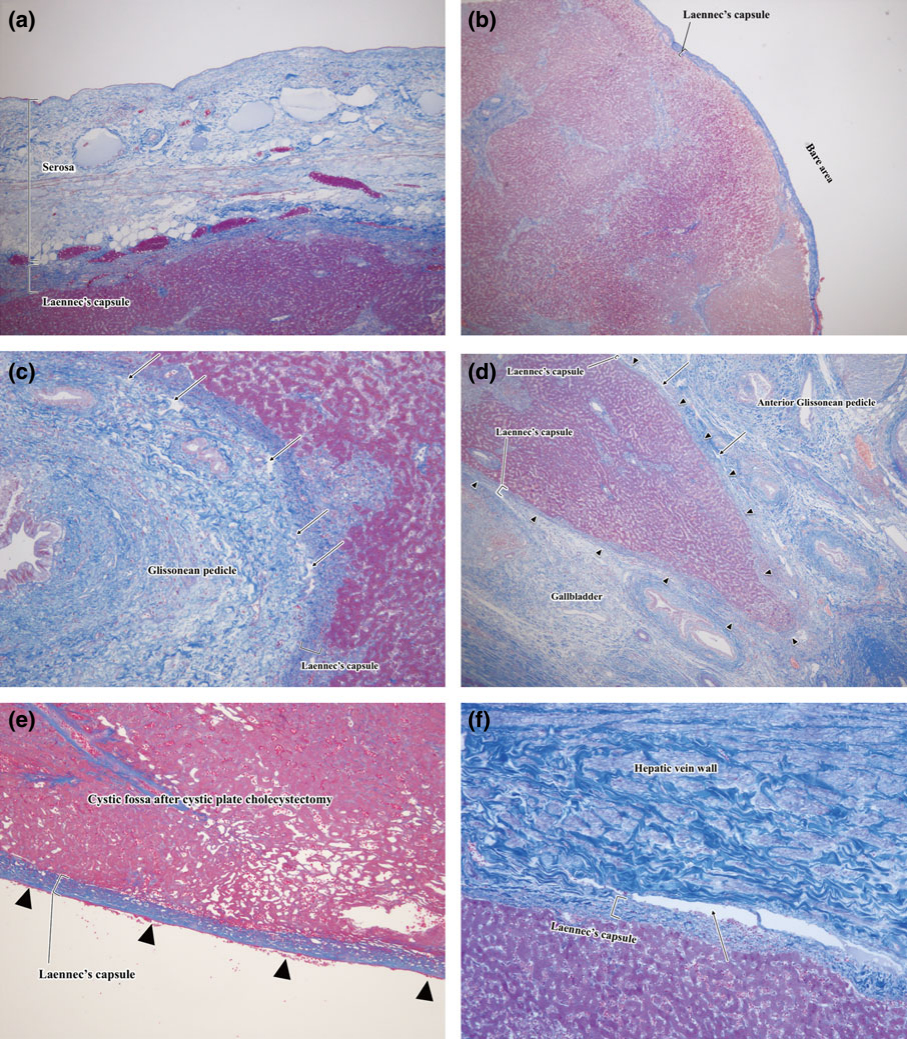
Histological findings of Laennec's capsule (Azan‐Mallory staining). (**a**) A dense fibrous layer (arrow head) was observed beneath the serosa. (**b**) The similar dense fibrous layer was also observed on the bare area. (**c**) In a cut surface of a resected specimen including a Glissonean pedicle, the similar fibrous layer was observed as a lining of the liver parenchyma surrounding the Glissonean pedicle with gaps (arrow). (**d**) A cut surface in the long axis direction of the anterior Glissonean pedicle revealed the similar fibrous layer extending from the surface of the anterior Glissonean pedicle to the liver bed indicating the layer attached to the liver parenchyma not to the Glissonean pedicle. (**e**) The similar fibrous layer was observed on the cystic fossa after cystic plate cholecystectomy. (**f**) The fibrous layer was also observed covering the hepatic vein wall with gap (arrow)

Regarding the history of the surgical anatomy of the liver, Walaeus first described the vasculo‐biliary sheath, which contains the portal vein, the hepatic artery, and the bile duct in 1640 14. Glisson also reported the Glissonean pedicle in 1642 15. Although some researchers had proposed a proper membrane of the liver, they confused it with the serosa or misunderstood as a nonexistent capsule of the Glissonean pedicles. In 1802, Laennec first described a proper membrane as the distinct structure from the serosa 16. Couinaud established the concept of the plate system as a fibrous thickening part of the Glissonean sheath 17, 18 and demonstrated that Laennec's capsule has no continuity with the Glissonean pedicle 3. He described detailed pathological findings on Laennec's capsule, but he did not give award to its importance 3 and Laennec's capsule had been ignored for more than 200 years. Recently, Hayashi et al. conducted the precise histological study of cadaveric livers with the elastic fiber and lymphatic vein staining and revealed that the so‐called Glissonean capsule was not derived from the Glissonean sheath but from Laennec's capsule surrounding the pedicles and that Laennec's capsule extended to the peripheral Glissonean pedicles 9. We also confirmed Laennec's capsule histologically as shown in Figure 2. Laennec's capsule could be observed as a dense fibrous layer beneath the serosa (Fig. 2a) and on the surface of the bare area (Fig. 2b). The similar fibrous layer also could be observed as lining of the Glissonean pedicle (Fig. 2c,d), on the cystic fossa after cystic plate cholecystectomy (Fig. 2e) and on the outside of the hepatic vein (Fig. 2f). In the process of standardizing liver resection, we came to believe that Laennec's capsule should be the essential structure for understanding the comprehensive surgical anatomy of the liver and standardizing the extrahepatic Glissonean pedicle isolation for anatomical liver resection. Herein, we proposed a novel comprehensive surgical anatomy of the liver based on Laennec's capsule.

## A novel comprehensive surgical anatomy of the liver based on Laennec's capsule

**Figure 3:**
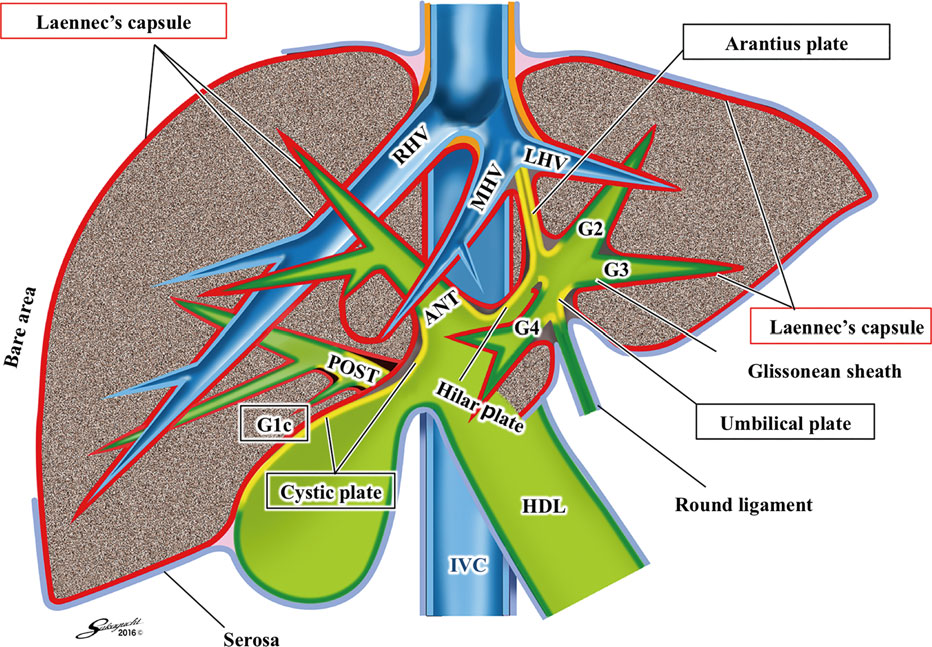
The schema of a novel comprehensive surgical anatomy of the liver based on Laennec's capsule**.** Laennec's capsule (*red and orange line*) covers not only the entire surface of the liver parenchyma beneath the serosa (*blue line*) including the bare area but also the intrahepatic parenchyma surrounding the Glissonean pedicles (*green line*) and the plate system (*yellow line*). Between the parenchyma of the cystic fossa and the subserosal layer of the gallbladder, Laennec's capsule and the cystic plate are interposed. There exists a gap (*gray space*) between Laennec's capsule and Glissonean pedicles (the plate system) that allows us to isolate the extrahepatic Glissonean pedicles. The red line between the peripheral Glissonean pedicle and the hepatic vein, and the orange line covering the suprahepatic inferior vena cava represent our hypothesis that the whole Laennec's capsule is a continuous structure originated from the septum transversum

Figure 1 shows the schema of a conventional anatomy of the liver; the bare area is covered by nothing, the parenchyma of the cystic fossa is adjacent to the subserosal layer of the gallbladder, the Glissonean pedicle is directly in contact with the intrahepatic parenchyma, and the hilar plate covers the outer circumference of the pedicles. On the other hand, Figure 3 demonstrates the schema of a novel comprehensive surgical anatomy of the liver; Laennec's capsule (*red line*) covers not only the entire surface of the hepatic parenchyma beneath the serosa (*blue line*) including the bare area but also the intrahepatic parenchyma surrounding the Glissonean pedicle surface (*green line*). Consequently, a gap exists between the Glissonean pedicle (the plate system) and Laennec's capsule (gray space) as shown in Figure 2 that allows us to isolate the extrahepatic Glissonean pedicle without parenchymal destruction. Because the plate system (*yellow line*) represents a fibrous thickened part of the Glissonean pedicle including vaginal ductuli 17, 18, 19, it would become a good landmark for the extrahepatic Glissonean pedicle isolation. Similarly, between the parenchyma of the cystic fossa and the subserosal layer of the gallbladder, Laennec's capsule and the cystic plate are interposed and the cystic plate also would become a good landmark. The red line between the peripheral Glissonean pedicle and the hepatic vein, and the orange line covering the suprahepatic inferior vena cava represent our hypothesis that whole Laennec's capsule is a continuous structure originated from the septum transversum as suggested by Sabourin 20. Actually such structures can be found during liver resection. Thus, this novel anatomy gives a theoretical background for the systematic extrahepatic Glissonean pedicle isolation. For standardization, it is required to establish the specific surgical techniques.

## Standardization of the systematic extrahepatic Glissonean pedicle isolation

**Figure 4:**
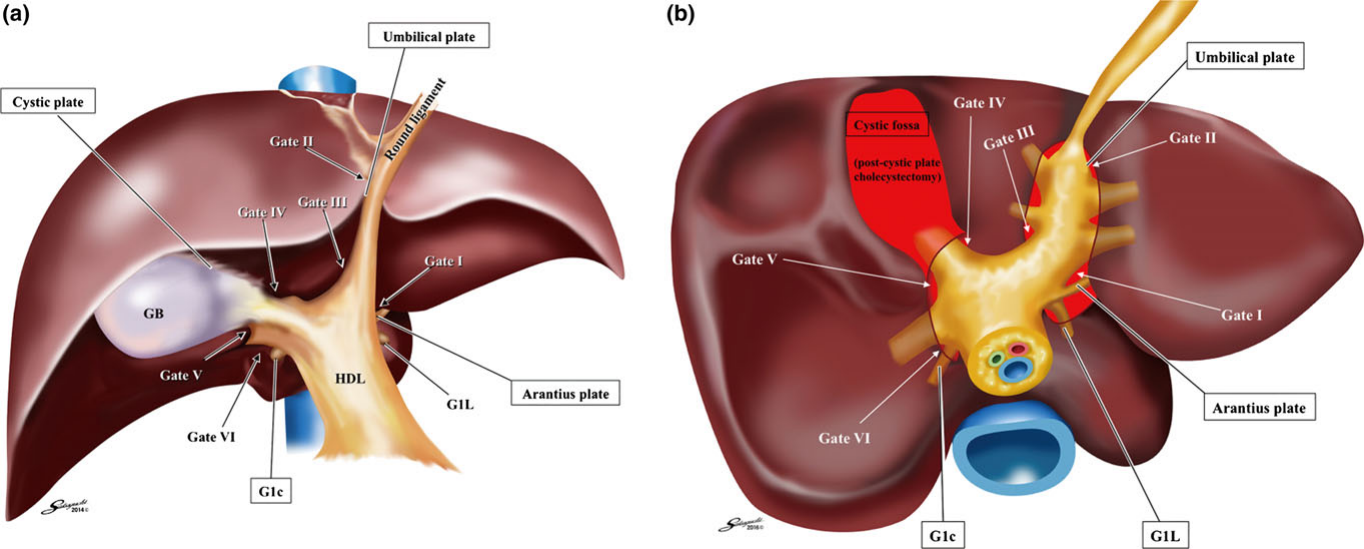
(**a**) The schema of the four anatomical landmarks and six gates in the frontal view. For standardization of the extrahepatic Glissonean pedicle isolation, it is essential to recognize the four anatomical landmarks (enclosed texts) and six gates. (**b**) The schema of the six gates and Laennec's capsule in the caudal view. The schema shows the relationship between the six gates and Laennec's capsule (*red area*). The gaps between Laennec's capsule and the Glissonean pedicle (the plate system) could be entered only at these six gates. **Gate I**: the caudal end of the Arantius plate, **Gate II**: the junction between the round ligament and the umbilical plate, **Gate III**: the right edge of the Glissonean pedicle root of the umbilical portion (*Gup*: G2 + 3 + 4), **Gate IV**: the left edge of the posterior extremity of the cystic plate or the anterior Glissonean pedicle, **Gate V**: the bifurcation of the right main Glissonean pedicle, **Gate VI**: the space between the posterior Glissonean pedicle and the G1c

For standardization of the systematic extrahepatic Glissonean pedicle isolation, it is essential to recognize the “four anatomical landmarks” and the “six gates (Gate I to Gate VI)” as shown in Figure 4a,b. The four anatomical landmarks are defined as follows: the Arantius plate, the umbilical plate, the cystic plate, and the caudate process pedicle (*G1c*). The gates are the points indicated by these anatomical landmarks. The well‐defined gaps between Laennec's capsule and the Glissonean pedicle (the plate system) could be identified and entered consistently only at the gates without parenchymal destruction and the extrahepatic Glissonean pedicle isolation could be standardized. By connecting the two gates precisely, the systematic extrahepatic Glissonean pedicle isolation could be achieved at will. Detailed surgical techniques are described below.

### How to isolate the extrahepatic Glissonean pedicles in the left liver

**Figure 5:**
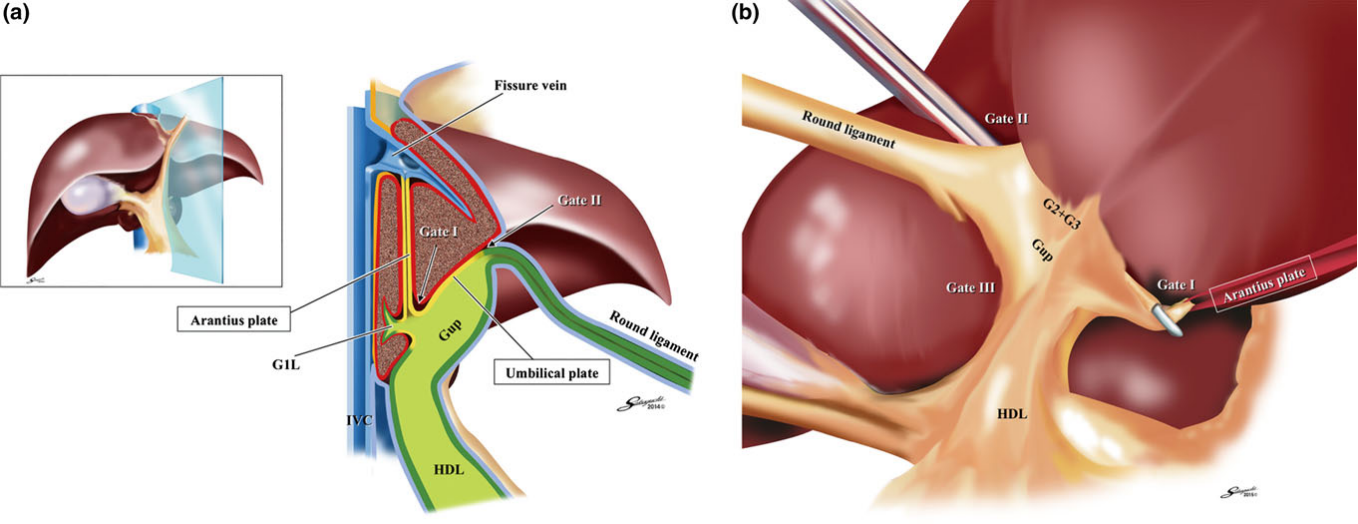
Extrahepatic Glissonean pedicle isolation in the left liver. (**a**) The vertical‐sectional image at the umbilical fossa. The schema demonstrates the boundary between the umbilical plate and Laennec's capsule covering the umbilical fossa. **Gate I** is identified as the caudal end of the Arantius plate, **Gate II** as the junction between the round ligament and the umbilical plate, and **Gate III** as the right edge of the Glissonean pedicle root of the umbilical portion (*Gup: G2 + 3 + 4*). (**b**) En bloc isolation of the G2 + 3. The common trunk of the Glissonean pedicle of segment 2 and 3 (*G2 + 3*) could be isolated en bloc by connecting **Gate I** and **II**. Thereafter, each pedicle could be isolated precisely

Figure 5a represents the vertical‐section at the umbilical fossa. The left Glissonean pedicle isolation should start from detaching the Arantius plate from Laennec's capsule to identify Gate I followed by detaching the umbilical plate from Laennec's capsule covering the umbilical fossa at Gate II. By connecting these two gates, the common trunk of the Glissonean pedicles of segment 2 and 3 (G2 and G3) could be isolated en bloc as shown in Figure 5b. Afterwards each extrahepatic Glissonean pedicle could be isolated precisely. The Glissonean pedicle of segment 4 (G4) or of the umbilical portion (*Gup*; G2 + G3 + G4) could be isolated connecting Gate II and III or Gate III and I in the same manner.

### How to isolate the extrahepatic Glissonean pedicles in the right liver

**Figure 6:**
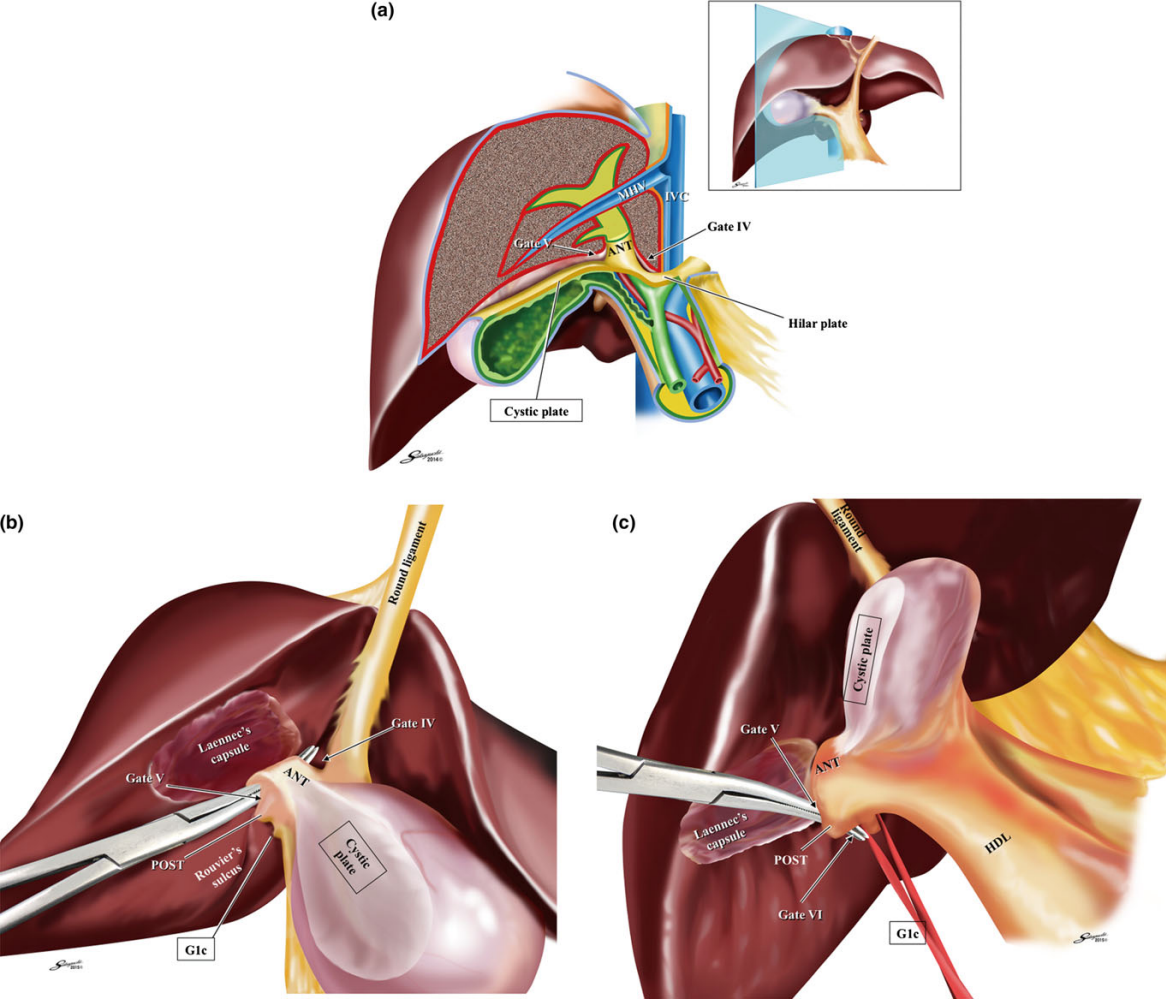
Extrahepatic Glissonean pedicle isolation in the right liver. (**a**) The vertical‐sectional image at the main portal fissure. The schema clearly demonstrates that the gap between the cystic plate and Laennec's capsule covering the cystic fossa leads to the precise layer of the right anterior Glissonean pedicle. (**b**) Isolation of the right anterior Glissonean pedicle. The right anterior Glissonean pedicle (*ANT*) could be reached by detaching the cystic plate from Laennec's capsule (“cystic plate cholecystectomy”) and isolated by connecting Gate **IV** and **V**. (**c**) Isolation of the right posterior Glissonean pedicle**.** The right posterior Glissonean pedicle (*POST*) could be isolated by connecting Gate **V** and **VI**

Figure 6a represents the vertical‐section at the main portal fissure. As clearly demonstrated, the right extrahepatic Glissonean pedicle isolation should start from detaching the cystic plate from Laennec's capsule covering the cystic fossa; we named the procedure as “the cystic plate cholecystectomy” that leads to the precise layer of the right anterior Glissonean pedicle and Gate IV and V could be identified. It is noteworthy that this procedure could isolate the Calot's triangle untouched even if the anomalous bile ducts are included. We can isolate the extrahepatic anterior Glissonean pedicle by connecting Gate IV and V as shown in Figure 6b and the extrahepatic posterior Glissonean pedicle by Gate V and VI as shown in Figure 6c. The G1c could be consistently identified at the entry of the Rouviere's sulcus. Further peripheral pedicles could be isolated extrahepatically using the subtraction method. However, it requires the utmost care not to dig into the intrahepatic parenchyma but to drag out the pedicles towards the hilum to prevent the unexpected injury to the hepatic veins.

## Future directions

Owing to the novel concept of comprehensive surgical anatomy of the liver based on Laennec's capsule, all the extrahepatic Glissonean pedicles including those of the caudate lobe could be isolated systematically, and thus, the hepatic inflow would be kept under controlled conditions during parenchymal dissection. The novel concept also could be applied to isolate and expose the main hepatic veins by preserving Laennec's capsule to the vein wall. Therefore, the anatomical liver resection could be standardized completely. In the near future, this concept would be expanded to any type of liver resection including laparoscopic or robotic liver resection and would bring innovative changes in the hepatobiliary surgery for spreading safe and curable liver resection.

## Conclusion

A novel comprehensive surgical anatomy of the liver based on Laennec's capsule would contribute to standardize the surgical techniques of the systematic extrahepatic Glissonean pedicle isolation for anatomical liver resection, and would bring innovative changes in hepatobiliary surgery for spreading safe and curable liver resection.

## Conflict of interest

None declared.
